# A Two-Stage EEG Microstate Fusion Framework for Dementia Screening and Alzheimer’s Disease/Frontotemporal Dementia Differentiation

**DOI:** 10.3390/bios16050258

**Published:** 2026-05-01

**Authors:** Lei Jiang, Yingna Chen, Yan He, Jiarui Liang, Xuan Zhao, Xiuyan Guo

**Affiliations:** 1 Fudan Institute on Ageing, Fudan University, Shanghai 200433, China; jianglei2@nbu.edu.cn; 2 Laboratory of Intelligent Home Appliances, College of Science and Technology, Ningbo University, Ningbo 315300, China; chenyingna@nbu.edu.cn (Y.C.); heyan@nbu.edu.cn (Y.H.); liangjiarui916@163.com (J.L.); zhaoxuan2026127@163.com (X.Z.); 3 MOE Laboratory for National Development and Intelligent Governance, Fudan University, Shanghai 200433, China

**Keywords:** EEG microstates, Alzheimer’s disease, frontotemporal dementia, multi-band feature fusion, task decoupling

## Abstract

Differentiating Alzheimer’s disease (AD) from frontotemporal dementia (FTD) using resting-state electroencephalography (EEG) remains clinically challenging because of their overlapping electrophysiological characteristics. Although EEG suits large-scale dementia screening, current method often overestimates performance because of epoch-level data leakage and multiclass feature competition in unified models. We propose a task-decoupled, two-stage hierarchical deep learning framework utilizing multiband EEG microstate dynamics. Continuous microstate sequences, modeled via Hungarian matching to preserve fine-grained temporal information, are processed using a normalizer-free 1D convolutional neural network (1D-CNN-NFNet) integrated with multi-head attention. By decoupling the workflow, Stage 1 performs generalized dementia screening using alpha and delta microstates, achieving an area under the curve (AUC) of 0.851. Stage 2 disentangles AD from FTD using delta and theta dynamics, yielding an AD-locking specificity of 86.1%. Evaluated under a strict subject-level leave-one-subject-out (LOSO) cross-validation protocol, the two-stage framework achieved 63.9% balanced accuracy, outperforming the single-stage baseline (55.4%) with a negligible inference latency of 0.733 ms. Furthermore, attention-based interpretability analysis links frequency-specific microstate alterations to underlying cortical disconnection syndromes. These results demonstrate that the framework provides a reproducible and interpretable auxiliary reference for dementia screening and subtyping in clinical neurology.

## 1. Introduction

### 1.1. Background

Dementia is a progressive neurodegenerative syndrome characterized by a gradual decline in cognitive function, frequently accompanied by synaptic dysfunction, disruption of large-scale brain network connectivity, and aberrant functional reorganization. According to the World Health Organization (WHO), more than 55 million people worldwide are currently living with dementia, with nearly 10 million new cases diagnosed annually [1,2]. As the global population ages, this epidemiological burden continues to escalate, posing unprecedented challenges for healthcare systems.

Among dementia subtypes, Alzheimer’s disease (AD) and frontotemporal dementia (FTD) are the two most prevalent. AD is characterized by episodic memory impairment, executive dysfunction, and language deficits, and is pathologically driven by β-amyloid deposition, tau tangles, and diffuse cortical neurodegeneration [3,4,5]. By contrast, FTD predominantly presents with early behavioral and personality changes or progressive language impairment, and its neuropathology is typically localized to frontotemporal cortical atrophy [6,7]. In the early stages of the disease, particularly in early-onset cases (onset < 65 years), the clinical manifestations of AD and FTD may overlap substantially, complicating diagnosis. Epidemiological studies indicate that in individuals aged 45–64 years, both early onset AD and FTD exhibit comparable prevalence rates of 10–20 per 100,000 [8]. Owing to the distinct prognostic trajectories and pharmacological management strategies, accurate and early differentiation between AD and FTD is of paramount clinical importance.

Currently, cerebrospinal fluid (CSF) biomarkers and positron emission tomography (PET) provide robust evidence of AD-related pathology [9,10]. However, their invasiveness, high cost, and limited accessibility restrict their routine use in large-scale screening in primary care settings. Therefore, non-invasive, cost-effective, and repeatable neurophysiological biomarkers capable of capturing large-scale brain network dysfunctions are needed.

Electroencephalography (EEG) offers millisecond-level temporal resolution and directly reflects the activity of the synchronized neuronal population. Studies have demonstrated that resting-state EEG in AD is characterized by a shift in spectral power toward lower frequencies (generalized slowing), reduced posterior alpha power (8–13 Hz), increased theta/delta activity, and decreased functional connectivity in higher-frequency bands [11,12,13]. However, conventional EEG analyses, such as power spectral density (PSD) or static coherence, rely largely on time-averaged representations, thereby failing to capture rapid, transient network reconfigurations at subsecond scales.

To characterize these transient dynamics, EEG microstate analysis has emerged as a powerful framework. Microstates are short-lived (approximately 60–120 ms), quasi-stable topographical configurations of scalp potentials that consistently cluster into four canonical classes (A–D). These topographies have been proposed as the electrophysiological correlates of large-scale resting-state networks [14,15,16]. Microstates serve as the fundamental building blocks of spontaneous cognitive processing by capturing rapid transitions between functional brain states.

Although microstate alterations have been reported in AD and related cognitive disorders, current diagnostic pipelines face three critical methodological limitations. First, information is lost through hard discretization. Traditional microstate pipelines convert continuous EEG signals into discrete label sequences via competitive backfitting [17]. This approach discards the soft spatial assignment information, thereby oversimplifying the fine-grained transitional dynamics and broadband masking effect during feature extraction. Most microstate studies rely exclusively on broadband signals (0.5–45 Hz) [18]. However, physiological oscillatory activities in distinct frequency bands reflect entirely different neurophysiological mechanisms (e.g., alpha for basal cognition and delta for structural atrophy). Mixing these frequencies often masks critical pathological features by high-frequency artifacts (e.g., electromyographic noise). The third limitation concerns the dilemma of multi-class feature competition. Existing state-of-the-art (SOTA) studies [19,20,21,22] predominantly attempted to train a single end-to-end model to simultaneously classify AD, FTD, and healthy controls (HC). This ambitious formulation forces the model to balance screening sensitivity and differentiation specificity within a shared feature space, inevitably leading to a diagnostic limitation in which highly overlapping phenotypes (as early FTD) are severely misclassified.

To address these gaps, we propose a Two-Stage Hierarchical Microstate Fusion Framework powered by a normalization-free deep neural architecture (1D-CNN-NFNet) for automated screening and differential diagnosis of dementia. Moving beyond traditional paradigms, this study explicitly decoupled diagnostic tasks to mirror real-world clinical workflows. The main contributions are summarized as follows:

- **Continuous Cross-Band Microstate Extraction:** We introduced a continuous spatial correlation representation that replaces discrete hard labels with template-wise similarity trajectories. Coupled with a Hungarian algorithm-based crossband alignment mechanism, this approach preserves sub-second dynamic richness and enables the systematic extraction of frequency-specific topographies.
- **Task-Decoupled Two-Stage Diagnostic Framework:** We developed a two-stage cascade architecture that eliminated multiclass feature competition. Stage 1 (Early Screening): feature-level fusion of the Alpha and Delta bands is used to maximize sensitivity in isolating patients with dementia from HC. Stage 2 (Differential Diagnosis): Delta–Theta fusion accurately differentiates the spatial atrophy patterns between AD and FTD, significantly outperforming traditional unified three-class models.
- **Physiologically Constrained Architecture Design:** To address the risk of overfitting in clinical EEG datasets, we designed a 1D-CNN-NFNet that integrates adaptive gradient clipping (AGC) and multi-head self-attention. This network uses microstate sequences to compress the feature space, achieving stable convergence without batch normalization. Furthermore, under a strict leave-one-subject-out (LOSO) cross-validation protocol, the framework supported leakage-free evaluation and demonstrated improved cross-subject generalization.

### 1.2. Related Work

Research on EEG-based dementia diagnoses has progressively shifted from static spectral descriptors to dynamic network-level models. Existing approaches can be broadly categorized into three main paradigms: (i) handcrafted-feature-based machine learning, (ii) deep learning-based representation learning, and (iii) microstate-driven dynamic modeling.

#### 1.2.1. Traditional Machine Learning Approaches in Dementia Recognition

Early EEG-based dementia classification studies predominantly relied on handcrafted features derived from spectral power, static coherence, entropy measures, and wavelet transforms [23,24,25]. These extracted features are typically fed into classical classifiers, such as support vector machines (SVM) [26], k-nearest neighbors (KNN) [27], decision trees [28], and ensemble models [29]. Although this traditional pipeline offers relatively strong interpretability and is suitable for small clinical datasets, it remains highly sensitive to subjective preprocessing choices, including referencing schemes, artifact rejection thresholds, and manual channel selection. Furthermore, feature distribution shifts across subjects can significantly impair the generalization capability of these models.

Importantly, inadequate cross-validation strategies in various early studies frequently led to inflated performance estimates. As demonstrated in [30], subject-level validation is critical in EEG-based AD classification, whereas epoch-level (or segment-level) random splits introduce data leakage; consequently, models memorize subject-specific artifacts rather than learn generalized pathological patterns. This highlights the need for rigorous subject-wise evaluation frameworks (e.g., LOSO) to support clinically meaningful generalizations.

#### 1.2.2. Deep Learning Approaches in EEG-Based Dementia Recognition

With the rapid advancements in deep learning, convolutional neural networks (CNNs), long short-term memory (LSTM) networks, and attention-based architectures have been increasingly applied to EEG classification tasks [30,31,32]. These models reduce reliance on handcrafted features by learning hierarchical representations directly from raw or minimally processed signals. Recent studies have reported highly competitive, or even near-perfect, performance in multiclass dementia classification using resting-state EEG [33].

However, as emphasized in [32], deep learning efficacy depends heavily on the dataset scale, input representations, and crucially, the validation protocol. Notably, many deep learning studies still rely on static spectrograms, band-power features, or statistical summaries as inputs [34], thereby primarily capturing time-averaged group differences rather than explicitly modeling rapid network-state transitions. Furthermore, from an architectural perspective, most existing SOTA deep learning frameworks [19,20,21,22] are designed as monolithic, single-stage classifiers. When optimizing a global loss function (e.g., categorical cross-entropy) across healthy controls and multiple dementia subtypes simultaneously, these networks often suffer from gradient dominance owing to easily separable classes (e.g., HC versus patients). Consequently, subtle, overlapping pathological boundaries, such as those between early-stage AD and FTD, are inherently under-optimized within a shared representation space. This architectural rigidity prevents the models from aligning with the hierarchical diagnostic pathways inherent in clinical practice, highlighting the urgent need for task-specific network decoupling.

#### 1.2.3. Microstates and Sequential Modeling in Dementia Recognition

To overcome the limitations of static analyses, EEG microstates have emerged as a powerful framework for characterizing fast, large-scale brain network dynamics [35]. Koenig et al. [36] established the methodological foundations and neurophysiological interpretations of microstates. Within the AD spectrum, several studies have reported altered microstate durations, coverage, and transition probabilities, highlighting their associations with cognitive decline and CSF biomarkers [15,37,38,39]. Tait et al. [39] and Ghassemkhani et al. [40] proposed microstate-complexity metrics and demonstrated that higher-order temporal structures provide discriminative information beyond classical static parameters. Compared with AD, microstate investigations of FTD remain scarce [41]. Nevertheless, emerging evidence suggests that microstate alterations closely correspond to the degradation of salience and frontotemporal networks, supporting the microstate-network-disease linkage hypothesis.

Despite promising advances, three key methodological limitations persist in current microstate-based diagnostic pipelines. (1) Information Loss via Hard Discretization. Traditional approaches convert continuous EEG signals into discrete label sequences (A–D) using competitive backfitting. This approach discards soft spatial assignment information and oversimplifies fine-grained transitional dynamics. (2) Under-exploration of Frequency-Specific Topologies. Most studies extract microstates exclusively from broadband signals (e.g., 0.5–45 Hz, 1–20 Hz), thereby ignoring that distinct frequency bands (e.g., alpha for thalamocortical integrity or delta for cortical deafferentation) encapsulate different pathological mechanisms. (3) Lack of Task-Decoupled Deep Sequential Modeling. The integration of microstate sequence representations with advanced deep learning frameworks is limited, particularly under strict, leakage-free subject-level validation for the challenging differential diagnosis between AD and FTD.

## 2. Materials and Methods

### 2.1. Dataset and Participants

This study utilized the public clinical EEG dataset OpenNeuro ds004504 [42], formatted according to the Brain Imaging Data Structure (BIDS) standard. The dataset comprised eyes-closed resting-state scalp EEG recordings from 88 subjects categorized into three cohorts: Alzheimer’s disease (AD, n=36), frontotemporal dementia (FTD, n=23), and healthy controls (HC, n=29). According to the original dataset descriptor, preliminary clinical diagnoses for all patients with AD and FTD were established based on the revised Diagnostic and Statistical Manual of Mental Disorders (DSM-III-R, DSM-IV) and ICD-10 criteria, combined with the NINCDS-ADRDA guidelines.

Subject demographics and Mini-Mental State Examination (MMSE) scores are listed in Table 1. Statistical analyses confirmed that the three groups were well-matched in age and sex distribution (p>0.05). This rigorous matching effectively controlled for potential confounding effects associated with normal aging, such as age-related brain slowing and physiological cortical atrophy. The MMSE scores reflect the cognitive status of each cohort: the HC group maintained normal cognitive function (30.0 ±0.0), whereas both the AD group (17.8±4.5) and the FTD group (22.2±8.2) exhibited significant moderate-to-severe cognitive impairment. Furthermore, no comorbid neurological conditions that could interfere with the electrophysiological signals were reported in the patient groups, thereby minimizing potential confounding factors in the extracted EEG features.

### 2.2. EEG Preprocessing and Data Augmentation

#### 2.2.1. Acquisition Parameters

**Figure 1:**
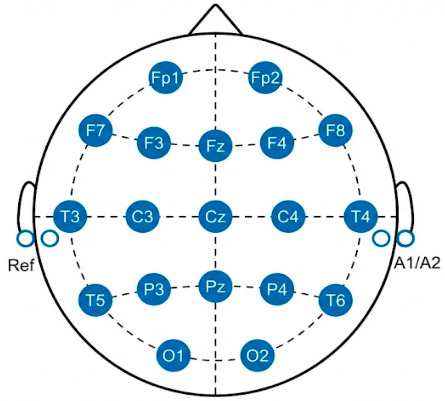
EEG electrode positions on the brain.

EEG recordings were acquired using a Nihon Kohden 2100 clinical device with 19 scalp electrodes placed according to the international 10–20 system shown in Figure 1.

Two mastoid electrodes (A1/A2) were used for impedance verification and as an offline reference. The sampling rate was set to 500 Hz with an acquisition resolution of 10 μV/mm. Recordings were obtained while the participants were seated in an eyes-closed, resting state.

#### 2.2.2. Preprocessing Pipeline

Signal preprocessing was executed in MATLAB (R2023a) using the EEGLAB toolbox (v2023.1), following the standardized pipeline provided in the dataset’s derivative documentation. The steps included: (i) Butterworth band-pass filtering between 0.5 and 45 Hz; (ii) re-referencing to the average of A1 and A2; (iii) Artifact Subspace Reconstruction (ASR) utilizing a conservative 0.5 s window and a standard deviation threshold of 17; (iv) Independent Component Analysis (ICA) via the RunICA algorithm to extract 19 independent components; and (v) automatic identification and rejection of eye-blink and muscle artifacts using the ICLabel classifier.

#### 2.2.3. Data Augmentation and Epoching

The first 5 min of artifact-free eyes-closed data were extracted from each subject to establish a standardized and computationally tractable input length for deep learning. This duration ensured consistent sampling while retaining sufficient stationary resting-state dynamics. The signals were then downsampled to 256 Hz to reduce computational overhead without compromising the frequency range of interest (≤45 Hz).

Data augmentation was performed using an overlapping sliding window epoching strategy. A window length of 10 s (yielding 2560 sample points per epoch) with a 5 s step size (50% overlap) was applied. This approach generated 58 partially overlapping epochs per subject (yielding a total of 5104 epochs across the dataset), substantially expanding the effective training sample size while preserving biologically plausible sub-second network transitions within each epoch.

### 2.3. Multi-Band Microstate Extraction and Continuous Spatial Correlation

Traditional microstate pipelines enforce hard discretization, thereby discarding the dynamic subtleties of the transitional periods between brain states, which is addressed by constructing a continuous spatial correlation sequence. Because dementia subtypes exhibit distinct oscillatory aberrations across different frequency bands, we systematically extracted microstate topologies across the canonical bands: broadband (0.5–45 Hz), delta (0.5–4 Hz), theta (4–8 Hz), alpha (8–13 Hz), beta (13–30 Hz), and gamma (30–45 Hz).

#### 2.3.1. GFP Peak Extraction and Cross-Band Template Alignment

Microstate templates were derived by topographical clustering at the peaks of Global Field Power (GFP). GFP quantifies the spatial standard deviation of the scalp potential field at a given time.

(1) $$GFP\left(t\right)=\sqrt{\frac{1}{N}\sum_{i=1}^{N}{\left(V_{i}\left(t\right)-\bar{V}(t)\right)}^{2}},$$

where N is the number of electrodes, $V_{i}\left(t\right)$ is the potential at electrode i at time *t*, and $\;\bar{V}(t)$ is the mean potential across all the electrodes at that instant. Because the GFP peaks represent moments of maximal signal-to-noise ratio and stable neural topographies, only these topographies were subjected to a Modified K-means clustering algorithm using a polarity-invariant spatial correlation metric. Consistent with the Global Explained Variance (GEV) criteria and prior literature, four canonical microstate classes (classes A–D) were identified, as shown in Table 2. To prevent data leakage, template extraction was performed dynamically within each LOSO fold using only the data of the training subjects.

Broadband templates were utilized as global anchors to address the label permutation problem arising from independent clustering across different frequency bands. We calculated the absolute spatial correlation matrix between the narrowband templates ($S_{j}$), and broadband anchors ($B_{k}$).

(2) $$C_{jk}=\left|\rho (S_{j},B_{k})\right|.$$

A cost matrix was subsequently defined as $D_{jk}=1-C_{jk}$. The Hungarian algorithm, a combinatorial optimization method for bipartite matching, was applied to solve the minimum-cost one-to-one assignment, ensuring that the physiological meaning of Classes A–D remained consistent across all evaluated frequency bands.

#### 2.3.2. Soft-Assignment Backfitting and Feature-Level Fusion

Instead of employing a winner-takes-all backfitting strategy, we calculated the polarity-invariant instantaneous correlation between the dynamic EEG topography x(t) and the aligned templates $m_{k}$:

(3) $$r_{k}\left(t\right)=\left|\rho (x\left(t\right),m_{k})\right|,k\in \left\{A,B,C,D\right\}.$$

For each 10 s epoch, continuous backfitting yielded a spatial correlation tensor of shape (4,2560), representing the soft-assignment trajectories of the four microstate networks. To support the proposed Two-Stage Hierarchical Diagnostic Framework, we employed feature-level fusion by concatenating the tensors along the channel dimension. As proposed and shown in Section 3.3, for Stage 1 (Early Screening), the Alpha and Delta sequences were fused to generate an input tensor of shape (8, 2560). In Stage 2 (Differential Diagnosis), the Delta and Theta sequences were fused to form the input tensor, enabling the network to focus purely on structural atrophy-related low-frequency dynamics.

### 2.4. Proposed 1D-CNN-NFNet Architecture and Validation Protocol

**Figure 2:**
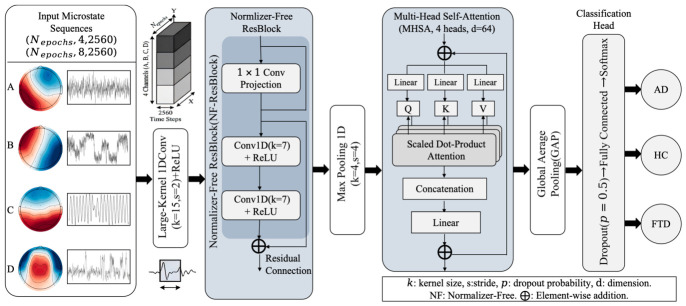
Proposed 1D-CNN-NFNet architecture.

To decode the pathological spatiotemporal dynamics embedded within the fused high-dimensional sequences, we designed a normalizer-free 1D CNN integrated with multi-head self-attention (1D-CNN-NFNet) (Figure 2).

#### 2.4.1. Residual Extraction and Self-Attention Fusion

The backbone contains a fused tensor with a batch size of 32. The initial layer utilizes a large-receptive-field 1D convolution (kernel size=15, and stride=2) to capture rapid microstate fluctuations across approximately 60 ms windows while performing preliminary downsampling. The core feature extractor comprised Normalizer-Free Residual Blocks (NF-ResBlocks) utilizing 1 × 1 projection shortcut and consecutive 1D convolution (kernel size=7) activated by the ReLU. Following strong downsampling via max-pooling (stride= 4), a multihead self-attention (MHSA) module (Heads=4, Embedding dimension=64) was embedded. The MHSA mechanism captures long-range temporal dependencies (e.g., the pathological cascading relationship between the dwell time of Class A and the subsequent activation of Class C). The representations were collapsed via Global Average Pooling (GAP), regularized by dropout (p=0.5), and mapped to binary logits for task-specific classification.

#### 2.4.2. Adaptive Gradient Clipping (AGC)

Given the inherently small batch sizes of medical datasets, traditional Batch Normalization (BN) can suffer from statistical oscillations. We completely removed the BN by adopting the NFNet paradigm and introduced Adaptive Gradient Clipping (AGC) to prevent gradient explosions. AGC evaluates the ratio of the gradient norm to the parameter norm on a unit-wise basis. For the *i–th* unit in layer l, the gradient $G_{i}^{l}$ corresponding to the weight $W_{i}^{l}$ is clipped according to

(4) $$G_{i}^{l}\left\{\begin{array}{c} \lambda \frac{max({\left\|W_{i}^{l}\right\|}_{F},\varepsilon )}{{\left\|G_{i}^{l}\right\|}_{F}}G_{i}^{l},\;\;if\;\frac{{\left\|G_{i}^{l}\right\|}_{F}}{max({\left\|W_{i}^{l}\right\|}_{F},\varepsilon )}>\lambda \\ G_{i}^{l},\;\;otherwise \end{array}\right.,$$

where ${\left\|\cdot \right\|}_{F}$ denotes the Frobenius norm, the clipping threshold λ is set to 0.04, and $\varepsilon =10^{-3}$ prevents zero-initialization deadlocks. This AGC mechanism effectively smooths the optimization landscape, ensuring stable convergence in the absence of normalization layers.

#### 2.4.3. LOSO Evaluation and Subject-Level Majority Voting

To eliminate the widespread data leakage caused by epoch-level splitting, this study followed the LOSO cross-validation protocol. In the 88-subject cohort, 88-fold cross-validation was used. In each fold, all epochs from a single subject were isolated as a completely unseen test set, whereas the remaining 87 subjects were split (8:2) into training and validation sets, respectively. The network was optimized using the Adam optimizer with a learning rate of $10^{-4}$. An early stopping mechanism was implemented to prevent overfitting and ensure optimal performance. Specifically, the training process was halted if the validation loss failed to improve for 20 consecutive epochs, thereby preserving the optimal model weights that exhibited the highest generalization capacity. Furthermore, a weighted cross-entropy loss function was adopted to mitigate the potential bias by class imbalance in the dataset.

Neurodegenerative EEG abnormalities exhibit dynamic temporal fluctuations. To enhance diagnostic robustness and alignment with patient-oriented clinical standards, we implemented a subject-level majority-voting mechanism. During inference, the 1D-CNN-NFNet independently predicted the class for all N epochs of the test subject. The final diagnostic label is determined by aggregating these predictions and assigning the subject to the class that receives most of the epoch-level votes. This aggregation strategy filters out transient noise, ensuring that the final confusion matrices and performance metrics evaluate the true generalized clinical utility of the models.

## 3. Results and Discussion

To ensure reproducibility and benchmark for computational efficiency, all experiments were conducted in a standardized environment. The hardware configuration included an NVIDIA GeForce RTX 4060 Laptop GPU (8 GB VRAM). The framework was implemented in Python 3.10 and PyTorch 2.5.1, using CUDA 12.2 for GPU acceleration. To guarantee deterministic results, all random seeds were set to 42. The full configuration details, including environment specifications and source code, are publicly available at https://github.com/winnile520-sys/AD-FTD-HC-data-and-code (accessed on 30 March 2026).

### 3.1. Results of Data Augmentation and Spatiotemporal Microstate Feature Extraction

**Figure 3:**
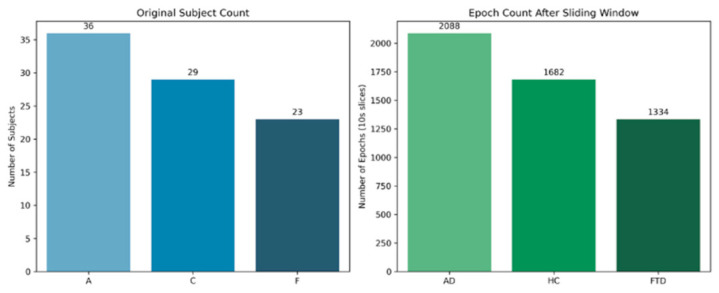
Comparison of sample sizes before and after signal resampling and sliding-window epoch extraction (Abbreviations: A = AD, Alzheimer’s disease; C = HC, Healthy Control; F = FTD, Frontotemporal dementia).

Following the rigorous preprocessing and overlapping sliding-window epoching strategy detailed in Section 2, eyes-closed resting-state continuous EEG recordings from 88 subjects were successfully augmented into 5104 high-quality, 10 s EEG epochs. Based on this, the extracted continuous spatial correlation sequences were reconstructed into a high-dimensional tensor with a shape of $\left(5104,\;4,\;2560\right)$. This augmentation strategy effectively preserved the high-order spatiotemporal transition characteristics of the microstate sequences and provided a robust, sufficient data foundation for the subsequent training and generalization of the 1D-CNN-NFNet deep learning model. The sample distribution across classes before and after augmentation is shown in Figure 3.

**Figure 4:**
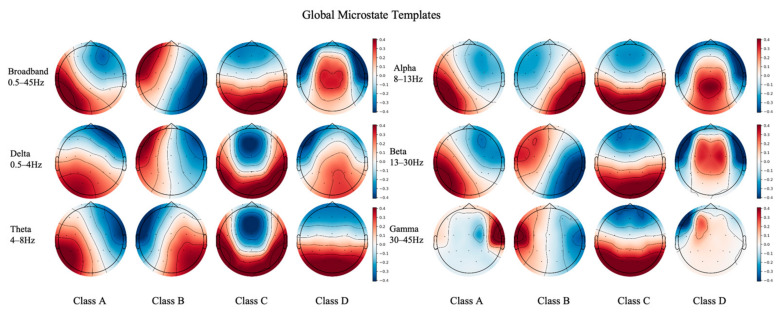
Extracted and spatially aligned topographies of the four canonical microstate classes (Classes A–D) across different EEG frequency bands.

During the feature extraction phase, the Hungarian matching algorithm introduced utilizes broadband (0.5–45 Hz) topography as a global anchor. This enabled the identification and forced crossband spatial alignment of the four canonical microstate classes (Classes A–D) across five independent rhythmic frequency bands: Delta, Theta, Alpha, Beta, and Gamma (Figure 4).

While demonstrating a high degree of global consistency, crossband microstate topographies also reveal specific rhythm-dependent fine-tuning phenomena. This verified the robustness of our alignment algorithm and the physiological validity of the extracted features.

#### 3.1.1. Cross-Band Consistency of Global Topologies

Across all valid frequency bands, the four microstate classes maintained their canonical core energy distribution patterns. The high-frequency beta band reflects active cognitive processing during wakefulness. For example, its topological structure closely matches that of the broadband anchor with clear boundaries, indicating that large-scale core brain networks possess high spatial stability in this state.

#### 3.1.2. Frontal Shift in Low-Frequency Slow Waves

In slow-wave bands, such as Delta and Theta, the core energies of Class C and D microstates exhibited a significant frontal shift, characterized by broader, smoother spatial gradients. Existing neurophysiological evidence suggests that overexpression of low-frequency slow waves typically reflects abnormalities in widespread cortical synchronization. Hyperactivity in the frontal regions is often directly associated with the pathological cascade of cognitive control decline and frontotemporal functional impairment [43].

#### 3.1.3. Occipital-Specific Activation in the Alpha Rhythm

Within the Alpha band, Class C microstate presented highly concentrated energy extrema in the posterior occipital region. According to the microstate theory in [44], microstate C is generally considered to be closely associated with the Default Mode Network (DMN) and the visual cortex. Because the Alpha rhythm during the eyes-closed resting state originates precisely from the posterior occipital visual areas, the precise alignment of spatial details further corroborates the reliability of the sequences extracted in this study.

#### 3.1.4. Noise Distortion in Marginal High-Frequency Bands

In the Gamma band, the energy extrema of Classes A and B were abnormally clustered toward the bilateral temporal poles. This phenomenon strongly suggests that this high-frequency band was inevitably contaminated by superficial scalp electromyographic (EMG) artifacts, resulting in severe distortion of its spatial topology. This observation provides a direct physical explanation of the subsequent performance degradation of the classification model when operating within this specific frequency band.

### 3.2. Performance Evaluation of the 1D-CNN-NFNet Framework

The diagnostic potential of the proposed model across distinct frequency rhythms was evaluated. Consequently, a scientific rationale for transitioning the diagnostic paradigm from a single monolithic multiclass differentiation to a task-decoupled hierarchical cascade architecture was established.

#### 3.2.1. Epoch-Level Frequency Band Sensitivity Evaluation

**Figure 5:**
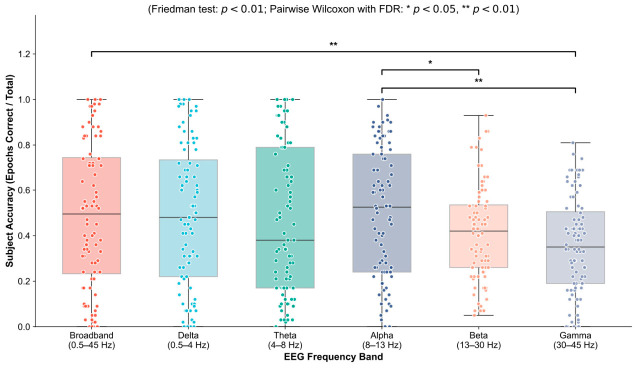
Subject-level classification accuracy distribution across six frequency bands.

To investigate the differential contributions of various frequency bands to the overall classification decision, we first compared the cross-validation accuracies at the epoch level across all 88 subjects (totaling 5104 epochs) (Figure 5). Statistical tests revealed highly significant global differences among the different EEG rhythms in their capacity to characterize dementia pathology (Friedman test, $\chi^{2}\left(5\right)=18.21,\;p=2.688\;\times 10^{-3}$).

Subsequent multiple comparison tests, corrected using the Benjamini–Hochberg False Discovery Rate (FDR) method, demonstrated the following:

1. Advantages of alpha bands
  The alpha band showed the highest median accuracy with relatively concentrated dispersion, significantly outperforming the beta ($p_{FDR}<0.05$) and gamma bands ($p_{FDR}<0.01$). The Alpha rhythm is highly dependent on the integrity of thalamocortical loops. During the progression of AD, the most typical early electrophysiological alterations are diffusing attenuation of alpha band power and slowing of its peak frequency [25]. The superior performance of the model in this band demonstrates that the 1D-CNN-NFNet can accurately capture the temporal dynamic abnormalities of microstates triggered by synaptic loss.
2. Representational advantages of low-frequency slow waves in characterizing organic brain network reorganization
  The median accuracy of the delta band (0.5–4 Hz) was high and did not differ significantly from that of the alpha band. From a clinicopathological perspective, low-frequency slow-wave activity is inhibited in healthy adults in awake, eyes-closed states. However, as AD and FTD progress, the apoptosis of cortical neurons and impairment of white matter tracts lead to severe cortical disconnection, which subsequently triggers abnormal bursts of pathological slow waves [45,46]. The outstanding performance of the model on delta-band microstate sequences confirms the high sensitivity of low-frequency large-scale networks to such structural-organic brain damage.
3. Failure of broadband and high-frequency features
  The Broadband (0.5–45 Hz) model, which integrates information across the entire frequency spectrum, did not achieve optimal results; its median was not higher than those of the isolated alpha or delta bands. This indicates that simply inputting full-band signals allows non-discriminative noise in higher frequency ranges to mask critical pathological features in low and middle frequency ranges. For instance, the gamma band, which is heavily contaminated by high-frequency EMG artifacts (with accuracies generally below 0.4), completely failed to provide valid topological patterns. This observation strongly underscores the need for targeted frequency-band selection before deep learning feature fusion.

#### 3.2.2. Subject-Level Decision Recognition Results Based on Majority Voting

**Figure 6:**
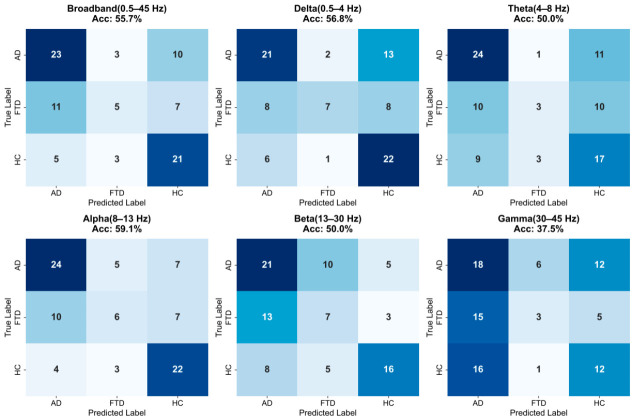
Subject-level confusion matrices of the proposed 1D-CNN-NFNet across six EEG frequency bands.

To avoid the artificially inflated performance commonly observed in epoch-level analysis of medical data and to align with the patient-centric principles of clinical diagnosis, we employed a majority voting mechanism to aggregate epoch-level predictions into a final subject-level decision. Figure 6 presents the final subject-level confusion matrices of the 1D-CNN-NFNet across the six evaluated frequency bands. The asymmetric misclassification patterns within these matrices highlight the differential advantages of specific frequency bands in handling distinct clinical diagnostic subtasks.

From an overall metric perspective, the alpha band performed best (accuracy = 59.1%, Macro F1 = 55.0%). More importantly, when considering the decision boundary between AD and HC exclusively, the alpha band identified 77.4% of patients with AD and 84.6% of healthy controls, suppressing the critical false-negative error (misdiagnosing AD as HC) to a remarkably low number of seven cases. This result indicates the pivotal role of the alpha band as a core biomarker for the preliminary screening of early cognitive decline.

When confronted with the core clinical challenge of differentiating AD from FTD, the Delta band exhibited exceptional discriminative potential. The confusion matrix showed that among the 36 patients with AD, the delta model misclassified only two cases as FTD. Pathologically, FTD is characterized primarily by the localized atrophy of the frontal and anterior temporal lobes, whereas AD presents with diffuse network degradation [47]. Because slow waves exhibit high spatial smoothness, the low-frequency delta band can capture the spatial discrepancies in these structural damage patterns more effectively than basal rhythms, thereby effectively preventing cross-confusion between dementia subtypes.

Furthermore, a common limitation across all frequency bands is that the classification decisions for samples with true FTD labels are highly dispersed. For example, in the alpha band, nearly 50% of patients with FTD were categorized as either HC or AD. This phenomenon corroborates the high heterogeneity and clinical variability of early-stage FTD in real-world clinical settings. Since early FTD primarily manifests as behavioral changes, its electrophysiological network phenotypes easily overlap with those of AD and severe late-life depression [44]. In addition, the limited FTD sample size (n=23) in this cohort restricted the depth to which single-band networks could learn particular topological regularities.

### 3.3. Proposal and Evaluation of the Cascaded Two-Stage Diagnostic Architecture

As demonstrated, the traditional end-to-end three-class framework suffers from multiclass feature competition during global gradient updating. Consequently, a monolithic model cannot simultaneously optimize the high sensitivity required for disease screening and the fine-grained specificity needed for subtype differentiation. However, the distinct complementary roles of different frequency bands in specific diagnostic subtasks provide critical insights for overcoming this classification limitation: the alpha band excels at distinguishing HCs, whereas the delta and theta bands excel at characterizing organic structural damage. Therefore, a two-stage cascaded diagnostic network was adopted.

#### 3.3.1. Stage 1: Early Cognitive Impairment Screening Based on Alpha-Delta Fusion

The primary stage of the hierarchical framework was designed to minimize the false-negative rate (omission of diagnosis) using the complementary neurophysiological properties of Alpha and Delta rhythms. Specifically, the alpha band serves as a sensitive indicator of baseline cognitive integrity, while the delta band is highly susceptible to pathological slow-wave intrusions during the prodromal phases of neurodegeneration. These frequency-specific microstate sequences were concatenated and an 8-channel fused feature tensor ($X_{fused}\in R^{8\;\times 2560}$) was reconstructed. This approach effectively transformed the screening task from a single-rhythm observation into a multi-dimensional analysis of large-scale brain network stability.

- Quantitative performance analysis

**Figure 7:**
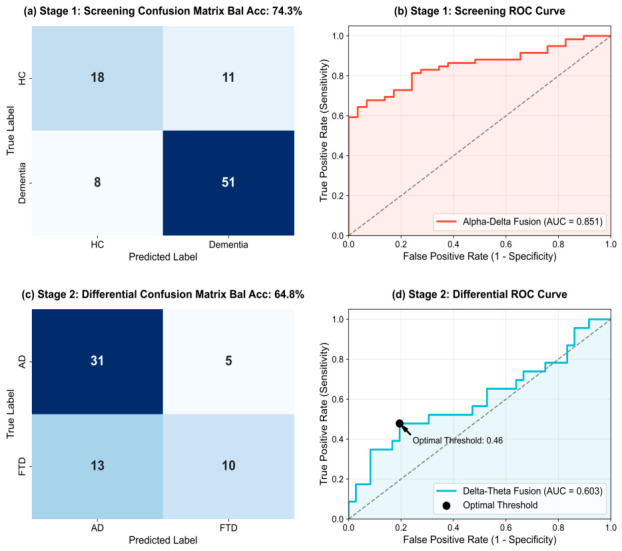
Performance evaluation of the proposed two-stage hierarchical diagnostic framework based on multi-band feature fusion. (**a**) Confusion matrix and (**b**) receiver operating characteristic (ROC) curve for Stage 1. (**c**) Confusion matrix and (**d**) ROC curve for Stage 2. The black dot on the ROC curve in (**d**) indicates the optimal operating threshold determined by Youden’s J statistic.

The Stage 1 framework was evaluated using the rigorous subject-level LOSO cross-validation, incorporating the entire cohort (88 subjects: 59 Dementia and 29 HC). The model achieved an area under the receiver operating characteristic curve of 0.851 and a balanced accuracy (defined as the arithmetic mean of class-specific recalls) of 74.3%, shown in Figure 7a,b. The screening module exhibited high clinical sensitivity, successfully identifying 88.9% (32/36) of AD patients and 82.6% (19/23) of FTD patients. FTD detection is particularly characterized by an insidious onset and high phenotypic overlap with other disorders. Therefore, the above-mentioned results underscore the efficacy of the joint Alpha-Delta network in capturing generalized cognitive degradation. In addition, these results establish a reliable benchmark for non-invasive, large-scale dementia screening.

- Neurophysiological interpretability

**Figure 8:**
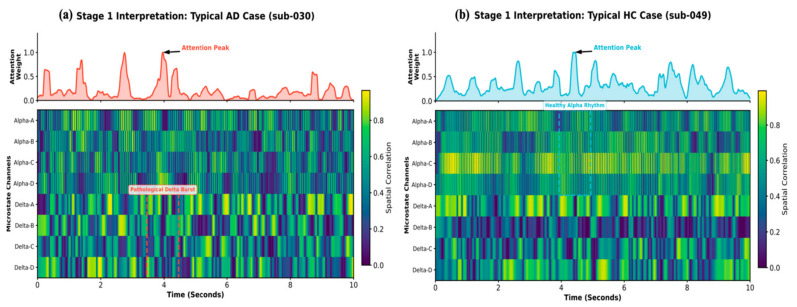
Neurophysiological interpretability of Stage 1 screening via MHSA attention maps. (**a**) Typical AD subject (sub-030, $P_{dementia}=1$) and (**b**) HC subject (sub-049, $P_{dementia}=0.015$). In each panel, top curves represent MHSA-derived temporal attention weights; bottom heatmaps display spatial correlation sequences for Alpha and Delta bands. Dashed boxes and arrows highlight the model’s precise localization of pathological Delta bursts in AD and preserved Alpha rhythms in HC.

We ascertained that the 1D-CNN-NFNet converges on biologically relevant features rather than incidental statistical noise. For this, the temporal attention weights were extracted from the MHSA module and projected onto the fused microstate heatmaps (Figure 8). This visualization facilitated a better understanding of the decision-making rationale of the model.

The model exhibited a radical paradigm shift in attention when processing a patient with severe AD (sub-030, $P_{dementia}=1$) (Figure 8a). The physiological heatmap revealed a significant “Alpha dropout,” characterized by the fragmentation and attenuation of high-frequency rhythms. Conversely, the Delta channels (bottom four rows) displayed transient, high-intensity vertical “bright spots” (pathological Delta bursts), indicating cortical disconnection and reduced synaptic efficiency. The highly impulsive attention curve (red) exhibited sharp peaks that were millisecond-synchronized with these low-frequency intrusions. Thus, the model successfully diagnosed AD by primarily detecting pathological slow-wave events.

The decision process for a representative HC subject (sub-049, $P_{dementia}=0.015$) is shown in Figure 8b. The heatmap revealed a stable and continuous spatial correlation within the Alpha band (top four rows), particularly in Classes C and D, which are associated with the maintenance of the DMN. The attention profile (blue curve) was relatively smooth, with peaks anchored to periods of sustained, quasi-stable Alpha rhythms. Thus, for healthy individuals, the model first verified the preservation of baseline cognitive syntax.

By decoupling the screening task into the Alpha-Delta fusion space, the model successfully transformed abstract neural weights into a visually verifiable diagnostic rationale. This interpretability aligns the deep learning decision process with the diagnostic intuition of neurologists, thereby fostering trust in AI-assisted diagnostic systems.

#### 3.3.2. Stage 2: Precise Dementia Subtype Differentiation Based on Delta-Theta Fusion

The objective of Stage 2 was to further delineate the subset of patients identified with cognitive impairment (Stage 1) into the specific pathological subtype (AD vs. FTD). At this juncture, the diagnostic space of the framework exclusively contained the AD (36 subjects) and FTD (23 subjects) cohorts. In this stage, the subtle topographical variations in cerebral atrophy were captured by leveraging a Delta-Theta fusion network ($X_{fused}\in R^{8\;\times 2560}$), and low-frequency slow waves were utilized as proxy biomarkers for regional neurodegeneration.

- Quantitative performance analysis

The Stage 2 network, designed to extract organic brain damage features, successfully differentiated AD from FTD. The model reached a balanced accuracy of 64.8% (Figure 7c,d). Under the strict majority voting framework, the module maintained a high specificity of 86.1% (31/36) in identifying AD, although FTD sensitivity was relatively lower at 43.5% (F1-score: 0.526). This performance profile reflects the inherent difficulty of the differential diagnosis, as generalized brain slowing in AD can mask the focal FTD features.

Despite these challenges, the proposed model still significantly outperforms traditional diagnostic tools, such as a standard three-class model achieving ~45% accuracy. Furthermore, the determination of an optimal operating threshold via Youden’s J statistic provided substantial flexibility for balancing sensitivity and specificity in clinical decision-making (Figure 7d). In summary, the task decoupling strategy is essential for eliminating multi-class feature competition; it also highlights the value of low-frequency microstate topological networks in differentiating diffuse cortical atrophy (AD) from localized frontotemporal atrophy (FTD).

- Neurophysiological interpretability

**Figure 9:**
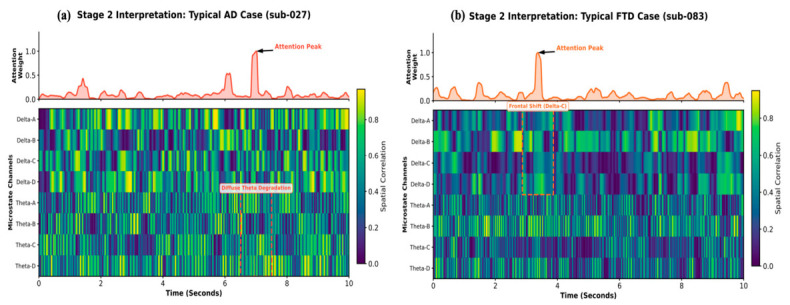
Neurophysiological interpretability of the Stage 2 differential diagnosis framework. (**a**) Typical AD subject (sub-027, $P_{FTD}=0.014$) and (**b**) FTD subject (sub-083, $P_{FTD}=0.999$). In each panel, top red/orange curves represent MHSA attention weights; bottom heatmaps visualize the Delta-Theta fused sequences. Dashed boxes and arrows highlight the diagnostic contrast between AD’s Diffuse Theta Degradation and FTD’s Frontal Shift.

To bridge the gap between algorithmic decision-making and clinical pathology, we analyzed the temporal attention distributions of the MHSA module for representative differential cases (Figure 9). By projecting attention weights onto the Delta-Theta fused microstate sequences, we visualized the divergent signatures differentiating AD from FTD.

For a typical AD subject (sub-027, $P_{FTD}=0.014$), the attention curve (red) exhibited a relatively distributed profile (Figure 9a). The underlying physiological heatmap revealed a widespread and fragmented activation pattern primarily localized within the Theta band (Theta A–D). In neuropathological terms, AD is characterized by a generalized failure of cortical networks and synaptic loss, leading to a diffuse slowing of the EEG spectrum. The framework differentiated AD from FTD based on the global accumulation of Theta-band anomalies instead of a single localized event. The absence of focal frontal bursts, combined with persistent spectral shifting in the Theta range, aligns with the generalized network failure typically observed in Alzheimer’s pathology.

A highly focused diagnostic pathway for the FTD subject (sub-083, $P_{FTD}=0.999$) is shown in Figure 9b. The attention curve (orange) exhibited sharp, impulsive peaks that selectively locked onto specific intervals. These peaks are millisecond-synchronized with high-intensity bursts on the Delta Class C channel. According to microstate topography, Class C is closely associated with the activity of the anterior cingulate cortex and frontal regions. The localized burst of slow-wave activity in this channel is caused by the Frontal Shift, indicative of frontotemporal lobar degeneration. While AD presents with generalized slowing, FTD exhibits more severe, localized neurodegeneration in the frontal lobes. The 1D-CNN-NFNet successfully exploited this spatiotemporal heterogeneity, identifying these focal Delta-C intrusions as specific evidence for FTD.

#### 3.3.3. Quantitative Group-Level Validation of Model Interpretability

Although Figure 8 and Figure 9 provide intuitive qualitative insights into the spatiotemporal features prioritized by the 1D-CNN-NFNet for representative subjects, the robustness of these mechanisms requires group-level statistical validation. To reduce potential selection bias from single-epoch visual anecdotes, we quantified the attention distribution across the entire dataset using the strict LOSO inference protocol. Specifically, the MHSA attention weights assigned to the target microstate sequences were aggregated across all 58 evaluation epochs for each subject to compute a subject-level attention allocation score.

**Figure 10:**
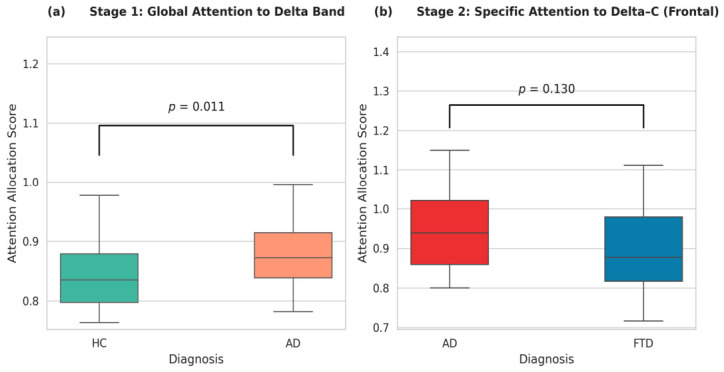
Group-level statistical validation of the MHSA attention allocation scores. Boxplots illustrate the distribution of attention weights assigned to targeted microstate sequences across all 58 evaluation epochs per subject. (**a**) Stage 1 screening: Normalized attention allocated to the global delta band in AD versus HC (p=0.011, Mann–Whitney U test). (**b**) Stage 2 differentiation: Attention allocated to the localized delta class C channel in FTD versus AD (p=0.130, Mann–Whitney U test).

A non-parametric Mann–Whitney U test was conducted to evaluate group-level differences in attention allocation (Figure 10). During Stage 1 screening, the normalized attention allocated to the delta band was significantly higher in AD patients than healthy controls (p<0.05), screening decisions rely on neurodegeneration-included generalized slowing rather than random artifacts. Furthermore, to verify that the randomly selected 10 s periods are not outliers, full-recording spectral histograms for the representative subjects were computed (Supplementary S3). These histograms confirm that the 10 s snapshots presented in Figure 8 align with the central tendency of the subjects’ overall neurophysiological states.

For the Stage 2 differentiation task, we evaluated the attention allocated to the delta class C channel. Although the FTD cohort exhibited a higher median attention score than the AD cohort, which aligns with the clinical expectation of frontotemporal lobar degeneration, the difference did not reach statistical significance (p=0.130). This result may reflect the inherent inter-subject variance of clinical EEG and the limited statistical power due to the small FTD sample size (N=23). Consequently, while the framework captures statistically significant generalized spectral shifts (Stage 1), consistently detecting highly localized focal anomalies (Stage 2) across a heterogeneous patient population remains challenging, highlighting the need for larger multi-center cohorts in future validation.

### 3.4. Ablation Study and Baseline Comparison

#### 3.4.1. Necessity of the Task-Decoupled Cascade Architecture

To validate the structural design of our framework, we established a performance progression from traditional machine learning to our proposed two-stage architecture. First, we evaluated Random Forest and Support Vector Machine models trained on canonical microstate statistical features, including occurrence, dwell time, coverage, and transition probabilities as detailed in Supplementary S1. As shown in Table 3, while these models achieved acceptable recall for HC, their FTD recall remained low at 17.4% and 30.4%, respectively. This resulted in overall balanced accuracy of 48.0% and 52.8%. These findings suggest that static features lack sufficient temporal resolution for complex subtyping tasks.

To address this limitation, we introduced the deep 1D-CNN-NFNet. When deployed as a single-stage 3-class classifier, the architecture improved AD recall to 63.9% and achieved a Balanced Accuracy of 55.6%. However, its FTD recall remained at 30.4%. Compared with the Stage 1 results in Section 3.3.1, where 82.6% of FTD patients were identified under a unified dementia class, this finding reveals a structural limitation. Simultaneous 3-class differentiation reduces FTD. Projecting the overlapping pathological signatures of AD and FTD into a shared diagnostic space introduces feature competition, making it difficult for the model to disentangle them.

By adopting the two-stage cascaded strategy, the framework effectively decouples generalized cognitive screening from fine-grained subtype differentiation. This approach mitigates feature competition by allowing specialized feature spaces for different diagnostic tasks, thereby enhancing AD recall to 86.1% and FTD recall to 43.5%. The overall 3-class balanced accuracy reached 63.9%, representing an 8.3% improvement over the single-stage model. To statistically validate this gain, we performed McNemar’s exact test on the subject-level predictions of the 59 dementia patients. The two-stage framework corrected 17 misclassifications made by the single-stage baseline while introducing only 6 new errors (p = 0.0347), confirming a statistically significant improvement in subtyping performance. Finally, benchmark testing on an NVIDIA RTX 4060 GPU showed that the total inference time for a 10 s EEG segment was 0.733 ms. Although this value was slightly higher than that of the single-stage model (0.364 ms), it remained well below the threshold for real-time clinical monitoring.

#### 3.4.2. Module-Level Ablation on Normalization and Attention Mechanisms

To evaluate the individual contributions of the core architectural components, we conducted a module-level ablation study focusing on the normalization strategy and global contextual modeling. In standard deep learning paradigms, batch normalization is widely used to accelerate convergence. However, clinical EEG microstate sequences exhibit profound inter-subject variability. Under the stringent LOSO cross-validation protocol, the test data represents an entirely unseen distribution. Consequently, the global running mean and variance accumulated by BN layers during training often fail to generalize to the novel test distribution, causing a statistical shift. To circumvent this, we adopted an NFNet backbone stabilized by AGC. Furthermore, while 1D-CNNs effectively extract localized morphological features, accurate dementia subtyping inherently relies on capturing long-range temporal dependencies and transition dynamics across the sequence. Therefore, a multi-head attention (MHA) module was integrated to establish global contextual representations.

To isolate the contributions of these modules, three architectural variants were evaluated on the single-stage 3-class task with batch size of 32, as shown in Table 4. To ensure a fair comparison, all variants maintained identical network depth, matched parameter counts, and identical training hyperparameters, with detailed layer configurations provided in Supplementary S2.

As shown in Table 4, Model A, where the NFNet blocks were replaced by conventional Conv-BN-ReLU sequences, yielded a balanced accuracy of 46.6%. Substituting batch normalization with the normalizer-free backbone in Model B mitigated the statistical shift induced by the LOSO protocol, improving the balanced accuracy to 49.6% and demonstrating enhanced cross-subject generalization. Finally, integrating the multi-head attention module in Model C enabled the framework to capture overarching temporal transition patterns, reaching 55.6%. In clinical translation, diagnostic inferences typically occur on a per-patient basis (batch size of 1). The NFNet architecture guarantees computational consistency between the training and single-sample inference phases, preventing the statistical biases inherent to batch normalization under such conditions. These ablation results indicate that the structural design is an effective optimization for neurophysiological signals.

### 3.5. Comparative Analysis with Existing State-of-the-Art Methods

To further evaluate the effectiveness and clinical translation potential of the proposed framework, we systematically compared our model with recent SOTA deep learning architectures and traditional clinical algorithms evaluated on the same public dataset (OpenNeuro ds004504) [42]. A comprehensive comparison is presented in Table 5.

As illustrated in Table 5, recent studies have reported classification accuracies of up to 88% [20]. However, these studies predominantly used epoch-level cross-validation. In this paradigm, highly correlated EEG segments from the same subject are distributed across both the training and testing sets, inadvertently introducing data leakage. Consequently, such results may overestimate the generalization ability of the models when deployed in real-world clinical settings. By contrast, our study adheres to a stringent subject-level LOSO protocol combined with majority voting. This approach aligns with the evaluation standards of the dataset publishers [42] and represents a realistic zero-shot test for clinical deployment. Under this rigorous protocol, our Stage 1 screening achieved an AUC of 0.851, outperforming the traditional LOSO-based spectral-index baselines (AUCs of 0.765 and 0.779) [21].

Furthermore, we compared our framework with the large-scale EEG foundation model, LEAD [22]. While LEAD achieved an accuracy 91.3%, it is crucial to note that this metric was attained on a binary screening task (dementia vs. HC), which corresponds to our Stage 1 objective rather than the full 3-class subtyping. Foundation models like LEAD require extensive pre-training on external datasets comprising thousands of subjects, incurring substantial computational overhead. In contrast, our proposed framework is notably lightweight and was trained entirely from scratch without external data dependency. This structural independence not only ensures the clinically negligible inference latency quantified in Section 3.4.1 but also provides a distinct deployment advantage in clinical settings where patient data privacy strictly prohibits the use of cloud-based large models.

Finally, regarding overall clinical feasibility, the proposed model extends beyond black-box predictions by offering a transparent neuropathological basis for its decisions. As discussed in Section 3.3, the strategic alignment of learned microstate features, such as mapping localized delta class-C intrusions to the frontal shift in FTD, and pathological delta bursts to the cortical disconnection syndrome in AD, significantly enhances the interpretability and reliability of the framework for clinical auxiliary diagnosis.

## 4. Conclusions

This study proposed a task-decoupled, two-stage hierarchical deep learning framework based on multiband EEG microstate dynamics for differentiating AD and FTD. By aligning the decision logic with established clinical diagnostic pathways, the proposed architecture effectively addressed the performance constraints of monolithic three-class models. Under a rigorous subject-level LOSO protocol, the framework achieved an overall balanced accuracy of 63.9%, representing a significant improvement over the 55.6% attained by the single-stage baseline, with a negligible inference overhead increase of only 0.369 ms.

Furthermore, comparative and module-level ablation studies validated the effectiveness of the core components. Utilizing an NFNet to mitigate statistical distribution shifts yielded a 3.0% performance gain, while the integration of multi-head attention to capture global temporal dependencies provided an additional 6.0% improvement. Specifically, the framework demonstrated a screening AUC of 0.851 in Stage 1 and an AD-identification specificity of 86.1% in Stage 2. The attention-based interpretability analysis provided neurophysiological insights into the decision-making process, such as the relevance of localized delta-band activity to pathological frontal shifts.

Despite these advances, this study is constrained by a limited sample size (N=88) and the inherent heterogeneity of clinical EEG signals. The results suggest that while the framework may serve as a valuable auxiliary tool, it is intended to support, rather than replace, comprehensive clinical evaluation. Future research will focus on validating these findings in larger, multi-center independent cohorts and exploring the integration of multimodal data, such as cognitive scales or structural imaging, to further enhance diagnostic robustness and generalizability.

## Figures and Tables

**Table 1:** Participant demographics and clinical characteristics.

Group	Subject (*n*)	Gender (M:F)	Age Years (Mean ± SD)	MMSE (Mean ± SD)
**AD**	36	13:23	66.4±7.9	17.8±4.5
**FTD**	23	14:9	63.6±8.2	22.2±8.2
**HC**	29	18:11	67.9±5.4	30.0±0.0

Abbreviations: AD = Alzheimer’s Disease; FTD = Frontotemporal Dementia; HC = Healthy Controls; M = Male; F = Female; SD = Standard Deviation; MMSE = Mini-Mental State Examination.

**Table 2:** Four canonical EEG microstate classes and their neurophysiological significance.

Microstate Class	Typical Topographical Features	Associated Functional Networks/Cognitive Processes
**Class A**	Right-anterior to left-posterior diagonal orientation	Auditory processing, language network, phonological perception
**Class B**	Left-anterior to right-posterior diagonal orientation	Visual system, visual imagery, spatial orientation
**Class C**	Anterior–posterior midline orientation	Salience network (SN), cognitive control
**Class D**	Symmetric posterior/parietal distribution	Default mode network (DMN), dorsal attention network (DAN), executive function

Abbreviations: SN = Salience Network; DMN = Default Mode Network; DAN = Dorsal Attention Network.

**Table 3:** Performance progression from traditional baselines to the proposed two-stage architecture (AD/FTD/HC).

Architecture	HC Recall	AD Recall	FTD Recall	Overall Balanced Accuracy	Inference Cost (ms)
Random Forest (0.5–45 Hz)	65.5%	61.1%	17.4%	48.0%	<0.05
SVM (0.5–45 Hz)	**72.4%**	55.6%	30.4%	52.8%	<0.05
Single-Stage (0.5–45 Hz)	**72.4%**	63.9%	30.4%	55.6%	0.364
Two-Stage (Proposed)	62.1% ^a^	**86.1%** ^b^	**43.5%** ^b^	**63.9%** ^c^	0.733

^a^ Obtained from Stage 1 (HC vs. dementia screening). ^b^ Obtained from Stage 2 (AD vs. FTD differential diagnosis). ^c^ The overall 3-class balanced accuracy. Note that the binary balanced accuracy for Stage 2 alone is 64.8%.

**Table 4:** Module-level ablation study evaluating normalization strategies and global context.

Model Variant	Normalization	Global Context	Balanced Accuracy	Key Characteristic
Model A (CNN-BN)	Batch Norm	None	46.6%	Relies on training batch statistics
Model B (NFNet)	NFNet+AGC	None	49.6%	Robust to inter-subject distribution shift
Model C (Proposed)	NFNet+AGC	MHA	55.6%	Captures global temporal dependencies

**Table 5:** Comparison with state-of-the-art (SOTA) studies on the same dataset.

Studies	Methods	Strategy	Tasks	Metrics
Miltiadous et al. [19]	DICE-net (CNN-Transformer)	LOSO	AD vs. HC	Accuracy: 83.3%
Alhaddad et al. [20]	CONECT+ML	10-fold CV	AD vs. FTD vs. HC	Accuracy: 88.0%
Pietrek et al. [21]	Spectral Indices (Alpha/Theta)	LOSO	AD vs. HC FTD vs. HC	AUC: 0.765 AUC: 0.779
Wang et al. [22]	LEAD (Large-scale EEG Model)	Subject-level	Dementia vs. HC	Accuracy: 91.3%
Ours	Two-stage 1D-CNN-NFNet	LOSO	AD vs. FTD vs. HC	Balanced accuracy: 63.9% Stage 1 AUC: 0.851; Stage 2 AUC: 0.603

## Data Availability

The public clinical EEG dataset used in this study is available at the OpenNeuro repository (dataset ds004504). The original contributions, data, and code presented in this study are openly available on GitHub at: https://github.com/winnile520-sys/AD-FTD-HC-data-and-code (accessed on 30 March 2026).

## Supplementary Materials

The following supporting information can be downloaded at: https://www.mdpi.com/article/10.3390/bios16050258/s1, Table S1: Hyperparameter specifications for baseline models; Table S2: Detailed specification layer for architectural variants; Figure S1: Consistency check between the selected 10-second snapshots and the whole-recording spectral distribution for representative AD subjects (sub-027 and sub-030); Figure S2: Consistency check between the selected 10-second snapshots and the whole-recording spectral distribution for representative HC and FTD subjects (sub-049 and sub-083).
