# Supplementary material for: A Two-Stage EEG Microstate Fusion Framework for Dementia Screening and Alzheimer’s Disease/Frontotemporal Dementia Differentiation
Source: Biosensors (Basel). 2026 May 1;16(5):258. doi: 10.3390/bios16050258 (PMC13204956; doi:10.3390/bios16050258)
Supplement: Supplementary file 1 [file biosensors-16-00258-s001.zip › biosensors-4266082-supplementary.pdf]

Supplementary Materials

Supplementary S1: Details of Traditional Machine Learning Baselines

S1.1 Microstate Feature Engineering

To ensure a fair comparison with the single-stage baseline, we extracted canonical microstate features from the broadband EEG signal (0.5-45Hz). For each 10-second segment, the following four metrics were calculated for each microstate class (A, B, C, D):

1. Occurrence (Occ), the average number of times a microstate class appears per second.
2. Duration (Dur), the average lifespan (in milliseconds) of a microstate class when it is active.
3. Coverage (Cov), the percentage of total recording time occupied by a specific microstate class.
4. Transition Probabilities (TP), the probability of transitioning from microstate i to microstate j, represented as P_{ij}.

The resulting 28-dimensional feature vector per subject was used as the input for the SVM and Random Forest baselines.

S1.2 Model Configurations and Hyperparameters

Both the SVM and RF models were implemented using the scikit-learn library. To ensure a fair comparison, we utilized a Grid Search with LOSO cross-validation to identify the optimal hyperparameters for each baseline.

Table S1. Hyperparameter specifications for baseline models.

Model	Hyperparameter	Search Space / Value	Final Optimized Selection
**Random Forest**	n_estimators	{100, 200, 500}	200
	Max Depth	{None, 10, 20, 30}	10
	Min Samples Split	{2, 5, 10}	5
	Criterion	{Gini, Entropy}	Gini
**SVM**	Kernal	{Linear, RBF, Polynomial}	RBF
	C(Regularization)	{0.1, 1, 10,100}	1.0
	Gamma	{Scale, Auto, 0.01, 0.001}	Scale
	Class Weight	{None, Balanced}	Balanced

S1.3 Detailed Performance and Subject-level Predictions(3-class)

1. Random Forest (0.5-45Hz, 3-class)

	Pred: HC	Pred: AD	Pred: FTD
True: HC (29)	**26**	3	**0**

True: AD (36)	6	**22**	8
True: FTD (23)	2	19	**2**

2. SVM (0.5-45Hz, 3-class)

	Pred: HC	Pred: AD	Pred: FTD
True: HC (29)	**25**	3	**1**
True: AD (36)	3	**23**	10
True: FTD (23)	2	17	**4**

3. Proposed Framework (0.5-45Hz, single-stage, 3-class)

	**Pred: HC**	**Pred: AD**	**Pred: FTD**
**True: HC (29)**	**18**	11	**0**
**True: AD (36)**	3	**31**	5
**True: FTD (23)**	2	11	**10**

Supplementary S2: Model Architectures and Implementation Details

S2.1 Detailed Layer Configurations for Ablation Variants

To ensure a fair comparison in the ablation study, Model A, B, and C maintained identical depth, kernel sizes, and feature dimensions. The core difference lies in the normalization strategy (BN vs. AGC) and the inclusion of the global context module (MHA).

Table S2. Detailed specification layer for architectural variants.

**Layer Type**	**Specification**	**Model A (CNN-BN)**	**Model B (NFNet)**	**Model C (Proposed)**
**Initial Conv**	$k = 15, s = 2$ 64 Ch	Conv1D + BN	Conv1D + AGC	Conv1D + AGC
**ResBlock1**	$k = 7, 1 \times 1$ shortcut, 128 Ch	NF-ResBlock + BN	NF-ResBlock + AGC	NF-ResBlock + AGC
**ResBlock2**	$k = 7, 1 \times 1$ shortcut, 256 Ch	NF-ResBlock + BN	NF-ResBlock + AGC	NF-ResBlock + AGC
**Down sampling**	Max-pooling ($stride = 4$)	Yes	Yes	Yes
**Context Module**	Global Temporal Modeling	Global Avg Pool	Global Avg Pool	MHSA (H=4, D=64)
**Classifier**	MLP + Softmax	Dropout (0.5)	Dropout (0.5)	Dropout (0.5)

S2.2 Training Hyperparameters and Optimization

- **Batch Size:** 32
- **Optimizer:** SGD with Nesterov Momentum (0.9)
- **Learning Rate:** Initial 1×10^{-3}, with Cosine Annealing scheduler
- **Weight Decay:** 2×10^{-5}
- **Dropout Rate:** 0.5

- **AGC Threshold (λ):** 0.01
- **Loss Function:** Label-Smoothed Cross-Entropy ($Smoothing\ factor = 0.1$)
- **Random Seed:** 42

Supplementary S3: Group-level Statistical Validation and Spectral Consistency

S3.1 Spectral Histogram Consistency Analysis

A key concern in clinical EEG interpretation is ensuring that selected short-term segments represent the patient's global neurophysiological state. To address this, we compared the spectral power distribution of the 10-second snapshots used for visualization (Figures 8 and 9) with the full-length (5-minute) resting-state recordings.

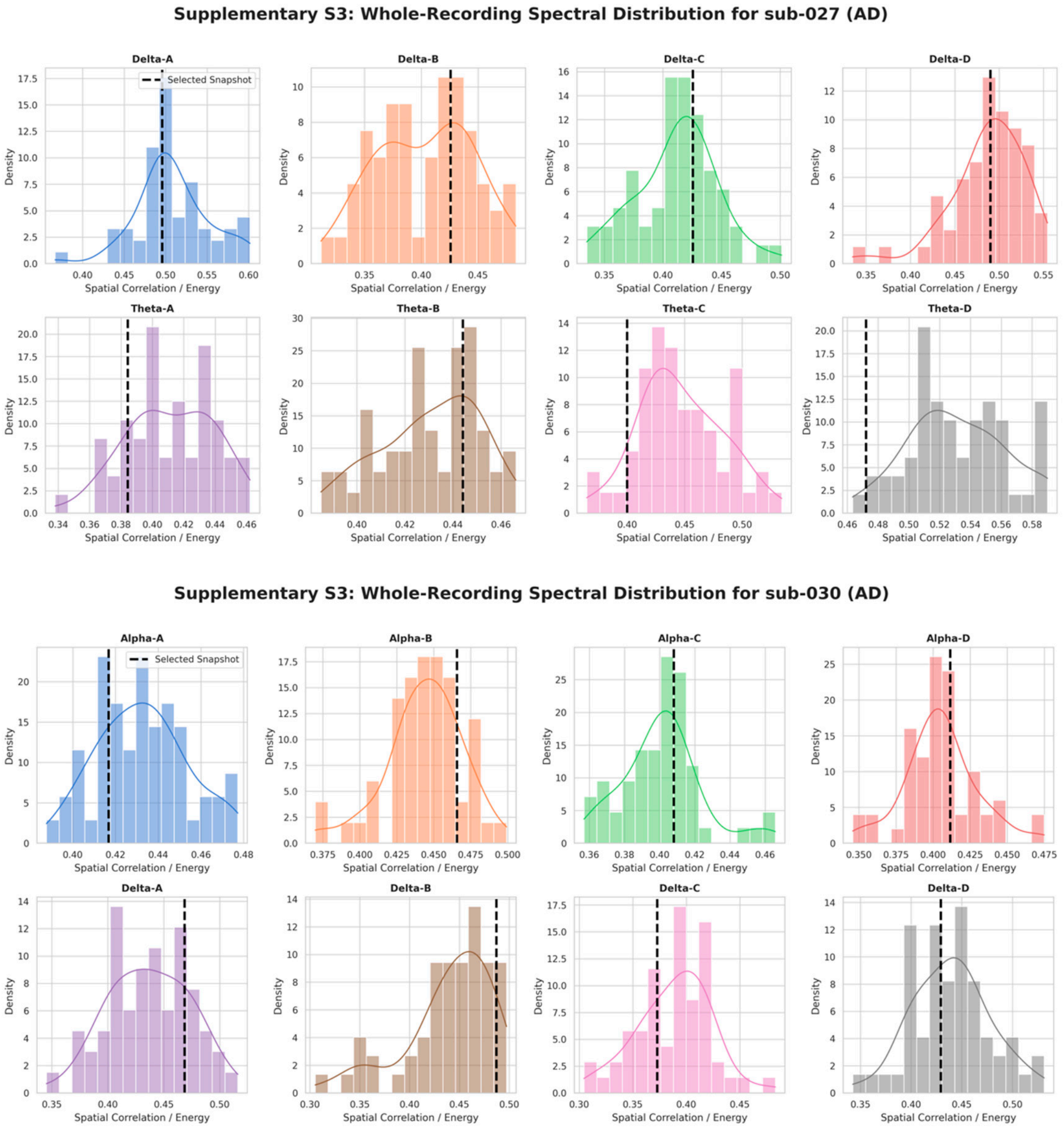

Figure S1. Consistency check between the selected 10-second snapshots and the whole-recording spectral distribution for representative AD subjects (sub-027 and sub-030).

As illustrated in Supplementary Figure S1, the selected snapshot (indicated by the black dashed vertical line) consistently aligns with the peak or the central mass of the

distribution across multiple frequency bands (Delta, Theta, Alpha) and microstate classes (A, B, C, D). For instance, in sub-027 (AD), the snapshot's correlation for the Delta-C and Delta-D components resides precisely at the mode of the 5-minute distribution. The histograms demonstrate that the neurophysiological patterns prioritized by our 1D-CNN-NFNet model (such as the pathological Delta Class-C intrusions) are not transient artifacts but are persistent features of the patient's brain activity throughout the recording. The snapshot's positioning within the high-density region of the histogram confirms that it represents the central tendency of the subject's neurophysiological profile. This consistency across different subjects (sub-027 and sub-030) and bands (from Alpha to Delta) substantiates the qualitative findings in the manuscript. It validates that the MHSA module effectively captures stable, long-range temporal dependencies that characterize the AD pathology, rather than random epoch-specific fluctuations.

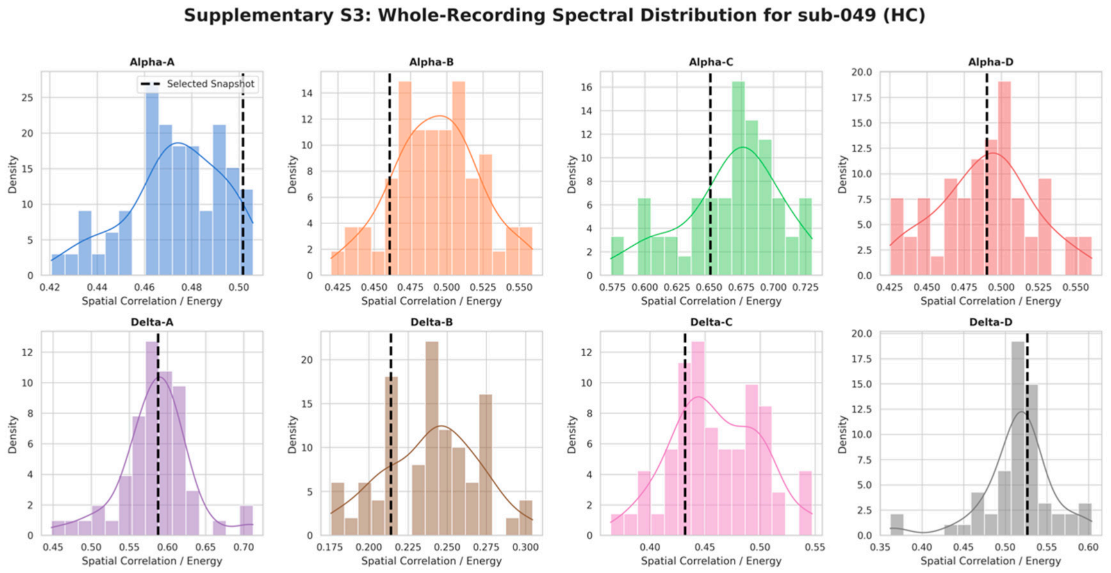

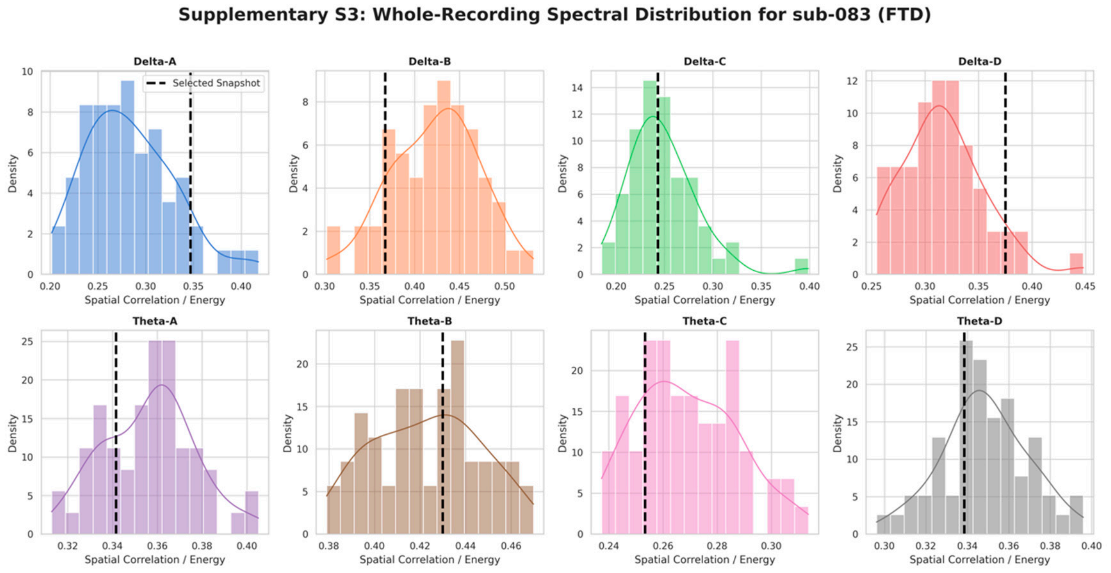

Figure S2. Consistency check between the selected 10-second snapshots and the whole-recording spectral distribution for representative HC and FTD subjects (sub-049 and

sub-083).

As illustrated in Supplementary Figure S2, for the HC subject, the selected snapshots in the Alpha and Delta bands consistently fall within the highest density regions of the 5-minute recording distributions. Specifically, the Alpha-A and Alpha-D snapshots are positioned near the peaks of their respective histograms. This confirms that the model's characterization of a normal EEG state is based on stable, rhythmic physiological activity rather than transient fluctuations. In the FTD subject, the snapshots for Delta and Theta bands (critical for FTD subtyping) align closely with the central mass of the global statistics. The alignment is particularly striking in the Delta-C and Theta-D channels, where the snapshot sits at the core of the pathological distribution. This validates that the focal anomalies identified by the 1D-CNN-NFNet for FTD patients (e.g., localized low-frequency intrusions) are representative of the subject's overall neurophysiological profile.

In summary, across all three diagnostic categories (HC, AD, and FTD), the selected 10-second snapshots consistently reflect the central tendency of the whole-recording spectral power and spatial correlation. This proved that the qualitative insights derived from our interpretability modules are representative and statistically grounded.
